# Trends in visual impairment among Austrian conscripts (1965-2003) after screening introduction: A retrospective, descriptive study

**DOI:** 10.1007/s00508-026-02750-8

**Published:** 2026-05-07

**Authors:** Lorenz Wassermann, Clemens Vass, Philipp Ken Roberts, Thomas Neumayer, Lin Yang, Thomas Waldhoer

**Affiliations:** 1 Department of Ophthalmology and Optometry, Medical University of Vienna, Vienna, Austria; 2 Klinik Landstraße, Vienna, Austria; 3 Departments of Oncology & Community Health Sciences, Cumming School of Medicine, University of Calgary, Hospital Drive NW, Calgary, Alberta Canada; 4 Department of Cancer Epidemiology and Prevention Research, Alberta Health Services, Holy Cross Centre, 2210-2nd Street SW, Box ACB, T2S 3C3 Calgary, AB Canada; 5 Department of Epidemiology, Center for Public Health, Medical University of Vienna, Kinderspitalgasse 15, 1090 Vienna, Austria

**Keywords:** Pregnancy passport examination, Austrian conscripts, Unilateral or bilateral visual impairment, Amblyopia, Strabismus

## Abstract

**Purpose:**

This retrospective descriptive study assesses the impact of Austria’s pregnancy passport (*Mutter-Kind-Pass, MKP*) program, which was implemented in 1974, on best-corrected visual acuity (BCVA), with a particular focus on its potential role in reducing the prevalence of unilateral or bilateral visual impairment (UoBVI).

**Methods:**

As part of mandatory military service, Austrian males aged 17–19 years undergo standardized medical examinations, including BCVA assessment for amblyopia detection and service eligibility. This study analyzed BCVA data from 1,694,681 conscripts born between 1965 and 2003 using data from the Austrian military conscription database. The UoBVI was classified according to defined BCVA thresholds (in Snellen decimal).

**Results:**

Among the total cohort, 1,561,292 conscripts (98.0%) demonstrated a BCVA of ≥ 0.8 in both eyes. The overall prevalence of amblyopia decreased from an initial 4% to 1.5% in the period between 1965 and 2003. When comparing individuals born between 1965–1967 and 2001–2003, those achieving BCVA ≥ 0.8 in both eyes increased from 147,002 (96.6%) to 80,419 (98.4%). Those with BCVA < 0.8 in both eyes decreased from 1511 (1.0%) to 383 (0.5%); individuals with BCVA between 0.4–0.7 in 1 eye remained relatively stable at 1432 (0.9%) vs. 719 (0.9%); while those with BCVA ≤ 0.3 in 1 eye decreased markedly from 2459 (1.6%) to 237 (0.3%).

**Conclusion:**

The MKP program, through structured ocular screening during key periods of visual development, appears to significantly contribute to the reduction in UoBVI prevalence in Austria, most likely due to early detection and treatment of amblyopia. These findings underscore the value of early vision screening as a component of public health strategy.

## Introduction

The introduction of the pregnancy passport examination program (*Mutter-Kind-Pass, MKP*) in Austria in 1974 marked a significant advancement in neonatal and pediatric healthcare. By linking participation to financial incentives, the program achieved a coverage rate exceeding 95% prior to 1996. Since its implementation, the MKP has been associated with notable reductions in perinatal and infant mortality, as well as a decline in the incidence of cerebral damage in children [12, 19, 20].

As part of this comprehensive screening program, several pediatric eye examinations are mandated: assessments by a pediatrician during the first week of life, between the fourth and seventh weeks and between the third and fifth months, followed by ophthalmologic evaluations at approximately 1 and 2 years of age [23]. These examinations aim to detect visually disabling, potentially blinding and in rare cases, life-threatening ocular conditions at an early stage, enabling timely and effective treatment. Early identification of ocular pathologies is particularly critical due to the time-sensitive nature of visual development. Disruption of normal visual input during this critical period can result in amblyopia, a neurodevelopmental disorder characterized by reduced best-corrected visual acuity (BCVA) in one or both eyes [14]. The most common cause of amblyopia is strabismus, followed by uncorrected refractive errors. Other contributing factors include congenital or acquired ocular conditions such as cataract, glaucoma, retinal disease, or structural malformations [10].

The MKP structured approach to early eye screening likely facilitates the prompt identification and management of these amblyogenic conditions, whether by pediatricians or ophthalmologists. This, in turn, may have contributed to an increase in pediatric referrals to ophthalmologists, greater public and professional awareness of childhood eye health, and enhanced interdisciplinary collaboration between healthcare providers and families, all of which are essential components of optimal pediatric care [21].

The present retrospective study investigates the long-term impact of the MKP program on the prevalence of unilateral or bilateral visual impairment (UoBVI), as determined by BCVA testing among male adolescents undergoing mandatory medical screening for military service in Austria. Given that most cases of visual impairment in this age group are presumed to be amblyopic in origin, this analysis offers indirect insights into the effectiveness of the MKP in reducing the national burden of amblyopia.

## Methods

### Study population

In Austria, all males reaching the age of 18 years in a given year are subject to compulsory military service and must undergo a mandatory medical examination to assess their fitness for service. Individuals with severe, permanent medical conditions that preclude participation in the examination are excluded. Detailed descriptions of the medical examination procedures have been published elsewhere [5, 8, 9, 22]. This study included all Austrian male conscripts with citizenship, born between 1965 and 2003, who underwent examination between the ages of 17 and 19 years. Age was recorded in complete years.

### Educational classification

Education was initially classified into four categories: low (lower than 9 years of compulsory school), medium (completed compulsory school), high (graduated from professional training or apprenticeship) and very high (qualified for university entrance). These categories were then collapsed into two groups: low to medium, and high to very high.

### Visual acuity assessment

As part of the medical examination all conscripts underwent visual acuity testing. Individuals with monocular or binocular best-corrected visual acuity (BCVA) below 0.8 decimal (equivalent to 20/25 Snellen) were referred for further evaluation by an ophthalmologist. These follow-up examinations included standardized BCVA assessments under photopic conditions, using Early Treatment Diabetic Retinopathy Study (ETDRS) logMAR charts (Good-Lite Company/Precision Vision, IL, USA) with 5% low-contrast sensitivity to minimize letter memorization. Testing was performed monocularly at a distance of 4 m [18].

### Refractive error assessment

Uncorrected non-cycloplegic visual acuity was measured using standard acuity testers (e.g., Maico, Germany; most recent model: Maico Titmus V4) or Snellen charts. If uncorrected acuity was below 0.8 (80/100), refractive error was assessed through either subjective refraction or confirmed using the conscript’s corrective prescription. In some cases, official ophthalmologic documentation provided by the conscript was used in lieu of BCVA testing [24]. Due to the retrospective nature of the dataset, the method and equipment used for measuring refraction likely varied over the study period and consistent data on measurement modalities are not available.

### Definition of visual impairment

The UoBVI was defined as both eyes showing a BCVA < 0.8 or 1 eye showing a BCVA < 0.8 and the other eye ≤ 0.8. The presence of UoBVI was further divided into visual groups with 1 eye 0.8 and the other eye 0–0.3 or 0.4–0.7.

### Statistical analysis

The prevalence of UoBVI was estimated as percentages with corresponding 95% confidence intervals (CI). Prevalence trends were analyzed over time, with the year 1974 (the MKP introduction) indicated by a vertical line in graphical presentations. Additional stratified analyses were conducted by educational level and Austrian province.

As this was a descriptive study, no formal statistical hypothesis testing was performed. All analyses were conducted using SAS version 9.4 (SAS Institute Inc., Cary, NC, USA).

### Ethics approval

The study design and conduct were approved by the Medical University of Vienna Ethics Review Board (ECS 1393/2018).

## Results

Data on 1,694,681 Austrian male conscripts born between 1965 and 2003, aged 17–19 years were analyzed. After excluding 6.0% of individuals with missing BCVA data, 1,592,495 conscripts were included in the final analysis. The distribution by age was as follows: 46.8% were aged 17 years, 48.5% were aged 18 years, and 4.8% were aged 19 years.

Across the entire study period, 1,561,292 conscripts (98.0%) demonstrated a BCVA of ≥ 0.8 in both eyes. A total of 8570 conscripts (0.5%) had a BCVA < 0.8 in both eyes. Additionally, 10,521 individuals (0.7%) had a BCVA between 0.4 and 0.7 in 1 eye with the other eye presenting a BCVA of ≥ 0.8, and 12,112 conscripts (0.8%) had a BCVA ≤ 0.3 in 1 eye while the other eye presented a BCVA of ≥ 0.8.

**Fig. 1:**
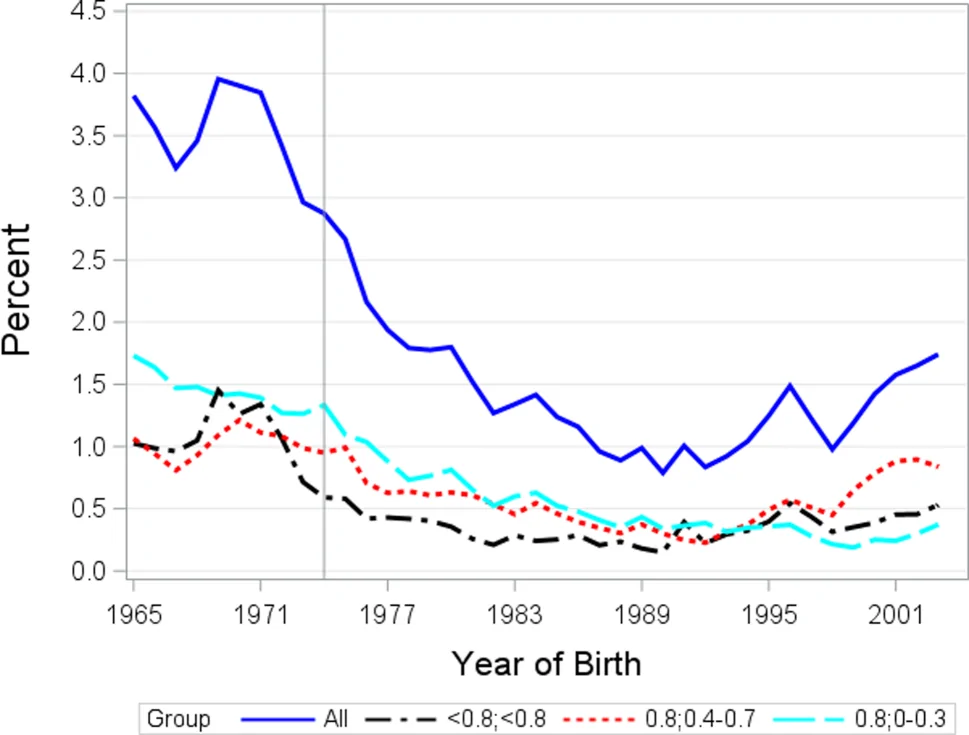
Time trend of prevalence of visual impairment in Austrian conscripts

Figure 1 illustrates the temporal trend in prevalence of amblyopia overall and by three specific categories. A U-shaped trend in the overall prevalence was observed, i.e., conscripts with 1 or 2 eyes with BCVA < 0.8, characterized by a strong decline from conscript born in 1964 to 1990, followed by a slight upward trend thereafter. The prevalence of conscripts with BCVA ≤ 0.3 in 1 eye showed a consistent decrease from approximately 1.6% to 0.3%. In contrast, the group with a BCVA between 0.4 and 0.7 in 1 eye exhibited a U-shaped trend, beginning and ending at around 0.9%. The proportion of individuals with a BCVA < 0.8 in both eyes was approximately 1.2% until 1970, after which it decreased to a trough of about 0.25% around 1990, followed by a slight increase to 0.5% at the conclusion of the study. Prevalence data for the periods 1965–1967 and 2001–2003 are presented in Table 1.

**Table 1:** Frequency and prevalence (95% confidence interval, CI) of visual impairment compared between 1965–1967 and 2001–2003

Visus group	1965–1967	2001–2003
*Both eyes ≥* *0.8*	147,002 96.64% (96.36–96.55)	80,419 98.36% (98.28–98.45)
*Both eyes <* *0.8*	1511 0.99 (0.94, 1.14)	383 0.47 (0.42–0.52)
*One eye 0.4–0.7*	1432 0.94 (0.89–0.99)	719 0.88 (0.82–0.94)
*One eye ≤* *0.3*	2459 1.61 (1.55–1.68)	237 0.29 (0.25–0.33)

**Fig. 2:**
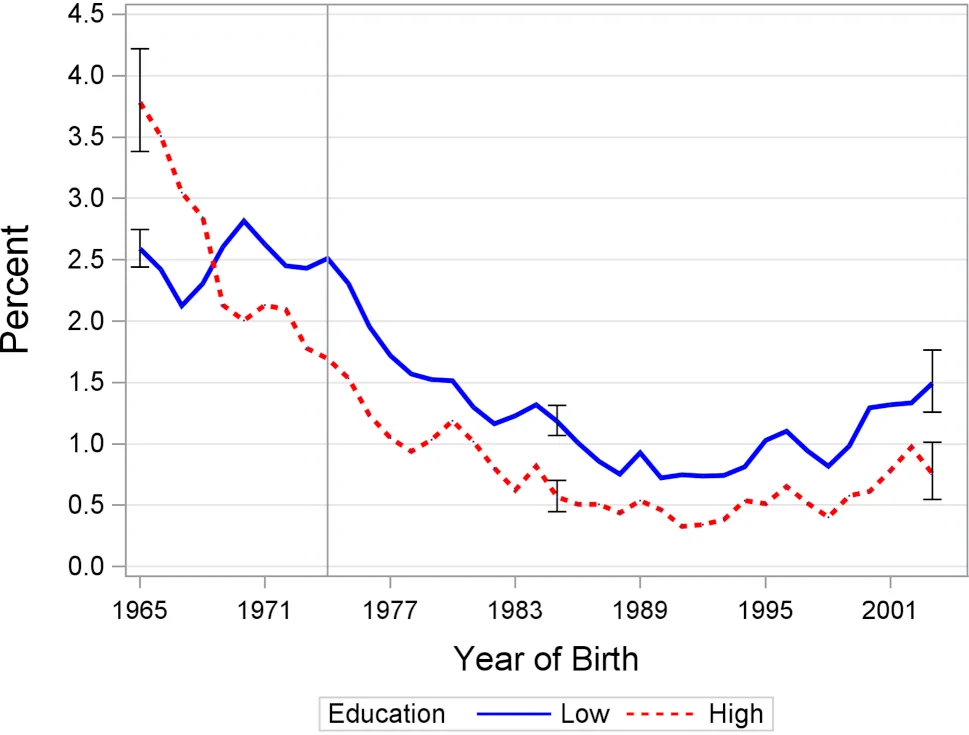
Prevalence of UoBVI (1 eye best corrected visual acuity 0.0–0.7) by education

The prevalence of UoBVI, combining the ≤ 0.3 and 0.4–0.7 BCVA groups, is presented by level of education in Fig. 2. Among conscripts with lower educational attainment, the prevalence remained constant until approximately 1974, after which a steep decline was observed until around 1996, followed by a slight increase thereafter. In contrast, the prevalence among conscripts with higher educational attainment was initially higher by approximately 1.1% during the first 3 years of observation; however, a sharp decline occurred and the prevalence remained consistently lower than that of the less educated conscripts by 0.4–0.5% for the remainder of the study period.

**Fig. 3:**
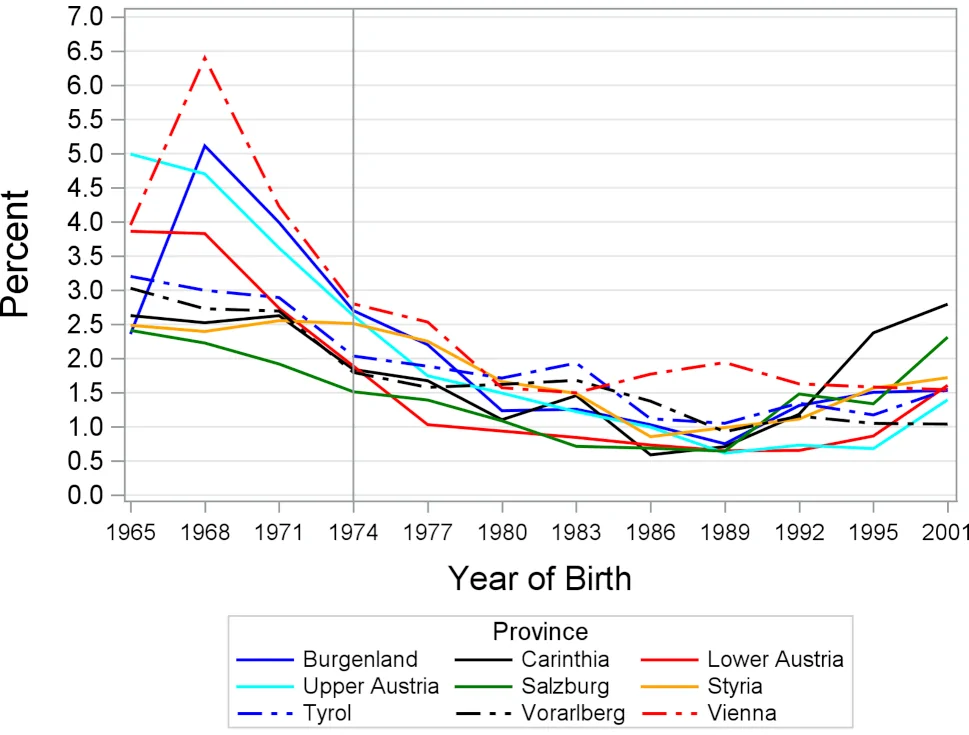
Prevalence of UoBVI (1 or both eyes best corrected visual acuity 0.0–0.7 by Austrian province)

Figure 3 shows the time trend in the prevalence of conscripts with BCVA < 0.8 in one or both eyes for each province in Austria. In almost all provinces, an increase in the prevalence was initially observed from 1965. From 1968 onwards, there was a fairly steep but continuous decline in the curve, and this trend continued from 1974 onwards. The only outliers here are Vienna until 1989, Carinthia in 1995 and Salzburg in 1995, with the remaining federal states settling at a very similar level between 1995 and 2001.

## Discussion

To the best of our knowledge, this study represents the largest retrospective descriptive analysis globally, comprising 1,561,292 individuals, to investigate the temporal trend in UoBVI using population-based data following the implementation of a national screening program.

Our retrospective analysis indicates a significant reduction in the prevalence of UoBVI and visual impairment across all evaluated groups from 1965–1967 to 2001–2003. Specifically, the prevalence of BCVA ≥ 0.8 in both eyes rose from 96.5% to 98.4%, the prevalence of BCVA < 0.8 in both eyes decreased from 1.0% to 0.5%, the prevalence of BCVA 0.4–0.7 in 1 eye remained stable at 0.9%, and the prevalence of BCVA ≤ 0.3 in 1 eye decreased from 1.6% to 0.3%.. Following implementation of the MKP, the overall UoBVI prevalence decreased from an initial rate of 4% to below 1% by 1987 and stabilized around 1.5% in 2001 (Fig. 1). This trend after the implementation of a screening program for UoBVI or amblyopia and the efficacy has been described previously [6, 11].

A systematic review by Mostafaie et al. [16], covering 131 studies between 1963 and 2019, reported a global amblyopia prevalence of 3.4% (95% confidence interval, CI 2.6–7.0%), with considerable regional variability, particularly in Africa. Other studies [2, 17, 25] report amblyopia prevalence rates between 2% and 3%, which are still higher than those observed in our study cohort.

Several studies predict an increasing prevalence of amblyopia in the coming decades. A meta-analysis conducted by Fu et al. [4] estimates the prevalence of amblyopia in 2019 with 99.2 million (95% CI: 71.7–146.1 million) and projects an increase in global amblyopia prevalence to 175.2 million (95% CI 81.3–307.8 million) by 2030 and to 221.9 million (95% CI: 83.7–429.2 million) by 2040; however, our study does not indicate a similar trend in Austria compared to the studies projections. The study of Fu et al. [4] reported varying prevalence rates across regions, including 2.90% in Europe, 2.41% in North America, 1.09% in Asia and 0.72% in Africa, with the highest amblyopia prevalence of 3.29% observed among individuals over the age of 20 years. The prevalence in children ranged from 1.0% to 6.0%, whereas in adults, it varied between 1.43% and 5.64% [15]. Our findings reveal a prevalence of amblyopia below 0.5% in both study groups in 2001 (BCVA 0.4–0.7 and 0.0–0.3), which is significantly lower than the prevalence rates reported in other studies. This further underscores the necessity of widespread screening programs for amblyopia.

The implementation of systematic screening programs for amblyopia remains a subject of ongoing debate, with variations in national recommendations. The U.S. Preventive Services Task Force (USPSTF) [14] advocates screening all children aged 3–5 years at least once for amblyopia or its risk factors, whereas the American Academy of Pediatrics and the American Association for Pediatric Ophthalmology and Strabismus [1] recommend initiating screening at birth. In contrast, the Institute for Quality and Efficiency in Health Care (IQWiG) [3] in Germany reported in 2008 that there was no conclusive evidence supporting the benefits of preschool vision disorder screening in children under 6 years of age.

A study conducted in Denmark by Høeg et al. [6] compared the prevalence of amblyopia between two cohorts: one cohort (born before 1959) that either received no screening or screening at 5 years of age and another cohort (born after 1974) that underwent screening at 4 years of age. A statistically significant reduction in amblyopia prevalence was observed in the preschool screened cohort (0.44%) compared to the nonpreschool screened cohort (1.78%, *p* = 0.024), corresponding to a fourfold reduction in amblyopia rates. These findings align with our study, which documented a decrease in prevalence from an initial 4% to between 0.5% and 1.5%.

In our study a continuous decline in UoBVI prevalence was also observed among participants with BCVA between 0–0.7 in 1 eye, with differences noted in prevalence based on parental educational attainment. In 1974, as the year of medical examination, the prevalence of UoBVI among participants of parents with lower educational attainment was 2.6%, compared to 1.6% in participants of parents with higher education. This gap persisted, although both groups exhibited a steady decline, stabilizing by 2003 at approximately 1.5% and 0.8%, respectively.

In a small-scale multicenter school-based study Wardati et al. [7] analyzed cross-sectional data on 480 children aged 7–12 years and reported no association of amblyopia prevalence with sociodemographic factors or parental educational attainment (*p* = 0.817). Zhang et al. [26] showed in the The Hong Kong Children Eye Study (HKCES), which included 4273 children aged 6–8 years, that no significant association was found between the occurrence of amblyopia and parental education (*p* = 0.413) or socioeconomic factors such as housing type or family income level; however, newly diagnosed cases of amblyopia were more prevalent among children of parents with lower education (*p* = 0.006) and those residing in low-cost accommodations (*p* < 0.001), findings consistent with our study.

These findings highlight the importance of public education, particularly targeting low-income families, to enhance awareness and uptake of pediatric vision screening programs.

The initiation of the MKP may have extended its impact beyond those directly screened, as siblings of MKP-screened children were also examined by pediatricians and ophthalmologists, potentially contributing to a broader reduction in undiagnosed amblyopia cases. Over time, healthcare providers’ diagnostic proficiency in screening methods (e.g., Hirschberg test, retinoscopy) improved, coupled with increased UoBVI awareness among healthcare providers and parents, enhanced further by strengthened interprofessional communication.

This study is not without limitations. A limitation of this descriptive study is that although visual acuity data from conscripts were analyzed on an individual level, it remains unclear whether individuals born after 1996, when financial incentives for MKP participation were modified, were systematically screened at birth. In a limited cohort of 200 children Lipp [13] reported an overall MKP screening participation rate of approximately 93% in 2012, with considerable variability based on child age and type of examination. Since 1987, visual screening has been recommended for children aged 10–14 months and optionally for those aged 22–26 months; however, for the latter group Lipp [13] documented a participation rate of only 47%, indicating a decline from the first to the second ophthalmological examination. The modification of financial incentives in 1996, a potential reduction in participation rates thereafter, and a slight increase in the amblyopia subgroup with BCVA 0.4–0.7 in 1 eye may further support the hypothesis of an association between MKP screening and amblyopia prevalence.

In conclusion, the MKP program, with its structured early ocular examinations conducted during critical periods of visual development, likely represents an effective measure in reducing amblyopia prevalence in later life. This impact is substantiated by both absolute and relative declines in UoBVI prevalence as well as improved collaboration between pediatricians and ophthalmologists, along with increased parental awareness regarding amblyopia detection. The efficacy of the MKP program likely extends beyond early screening alone, with the accumulating clinical expertise of healthcare providers within the MKP framework representing a crucial factor in enhancing early diagnosis and management of UoBVI.

### Key points

This study analyzed BCVA data from 1,694,681 conscripts born between 1965 and 2003.

The overall prevalence of visual impairment decreased from an initial 4% to 1.5% in the analyzed period.

The findings underscore the value of early vision screening as a component of public health strategy.

## Data Availability

The data supporting this study are not publicly available. Requests for access will be considered on a case-by-case basis and may be declined due to privacy, ethical, or legal restrictions.
